# Antibiotics for cancer treatment: A double-edged sword

**DOI:** 10.7150/jca.47470

**Published:** 2020-06-28

**Authors:** Yuan Gao, Qingyao Shang, Wenyu Li, Wenxuan Guo, Alexander Stojadinovic, Ciaran Mannion, Yan-gao Man, Tingtao Chen

**Affiliations:** 1 National Engineering Research Center for Bioengineering Drugs and the Technologies, Institute of Translational Medicine, Nanchang University, 1299 Xuefu Road, Honggu District, Nanchang, 330031 People's Republic of China.; 2 Queen Mary School, Nanchang University, Nanchang, Jiangxi 330031, PR China.; 3 Department of Pathology, Hackensack University Medical Center, 30 Prospec Avenue, Hackensack, NJ 07601, USA.; 4 Department of Pathology, Hackensack Meridian School of Medicine at Seton Hall University, 340 Kingsland Street, Nutley, NJ 07110, USA.

**Keywords:** Antibiotics, Cancer, Intestinal microbiota disorder, Cancer therapy

## Abstract

Various antibiotics have been used in the treatment of cancers, via their anti-proliferative, pro-apoptotic and anti-epithelial-mesenchymal-transition (EMT) capabilities. However, increasingly studies have indicated that antibiotics may also induce cancer generation by disrupting intestinal microbiota, which further promotes chronic inflammation, alters normal tissue metabolism, leads to genotoxicity and weakens the immune response to bacterial malnutrition, thereby adversely impacting cancer treatment. Despite the advent of high-throughput sequencing technology in recent years, the potential adverse effects of antibiotics on cancer treatments via causing microbial imbalance has been largely ignored. In this review, we discuss the double-edged sword of antibiotics in the field of cancer treatments, explore their potential mechanisms and provide solutions to reduce the potential negative effects of antibiotics.

## Introduction

Cancer is a common and frequently-occurring disease which seriously endangers human health. According to the view of modern cell biology, its basic mechanisms are abnormal growth and migration of cells with uncontrolled cell cycle, continuous self-renewal and reproduction of cancer stem cells 1. More than eight million people die of cancers each year, which places severe burden on economic and social development around the world 1.

Nowadays, weapons to fight cancers include surgery, radiotherapy, chemotherapy, immunotherapy and targeted therapy 2. Surgery alone has been offering a cure for cancers for centuries. With advancement in modern therapy, approximately 50% of all cancer patients are treated with radiotherapy because of the relative light damage to the body 2. However, surgery and radiation therapy can only be used to treat malignant cancers, which are confined locally to a particular organ 2. With the paradigm shift in our understanding of cancer as a systemic disease, chemotherapy and targeted therapy, which are used to kill cancer cells that have metastasized to distant sites in the body, have assumed increasingly larger role in cancer treatment and the challenges to patient care poised by acquired resistance and/or the the genotoxic nature of such treatments have started to come more sharply into focus 3.

Antibiotics refer to the secondary metabolites produced by microorganisms (including bacteria, fungi, actinomycetes) or higher animals and plants in the course of life that have anti-pathogen or other activities and can interfere with the development of other living cells 4. According to research findings, antibiotics can promote cancer apoptosis, inhibit cancer growth and prevent cancer metastasis. For these reasons, antibiotics are increasingly being used to assist in the treatment of cancers 5. However, the administration of antibiotics can also indiscriminately kill advantageous bacterial groups, such as *Lactobacillus* and *Bifidobacterium*, in addition to the pathogenic bacteria 6. The intestinal microbiome plays a very important role in cancer treatment. Thus, the use of antibiotics not only leads to disruption of the microbiome, but also reduces the body's immune capacity and promotes inflammation, which ultimately may impact and reduce the effect of cancer treatment 7.

Given the double-edged sword of antibiotics in the development of cancer therapy, this review aims to explore the role of antibiotics in cancer development and treatment, hoping to provide a better direction and method for the use of antibiotics in the treatment of cancer diseases in the future.

## Definition and Classification of Anticancer Antibiotics

**Figure 1:**
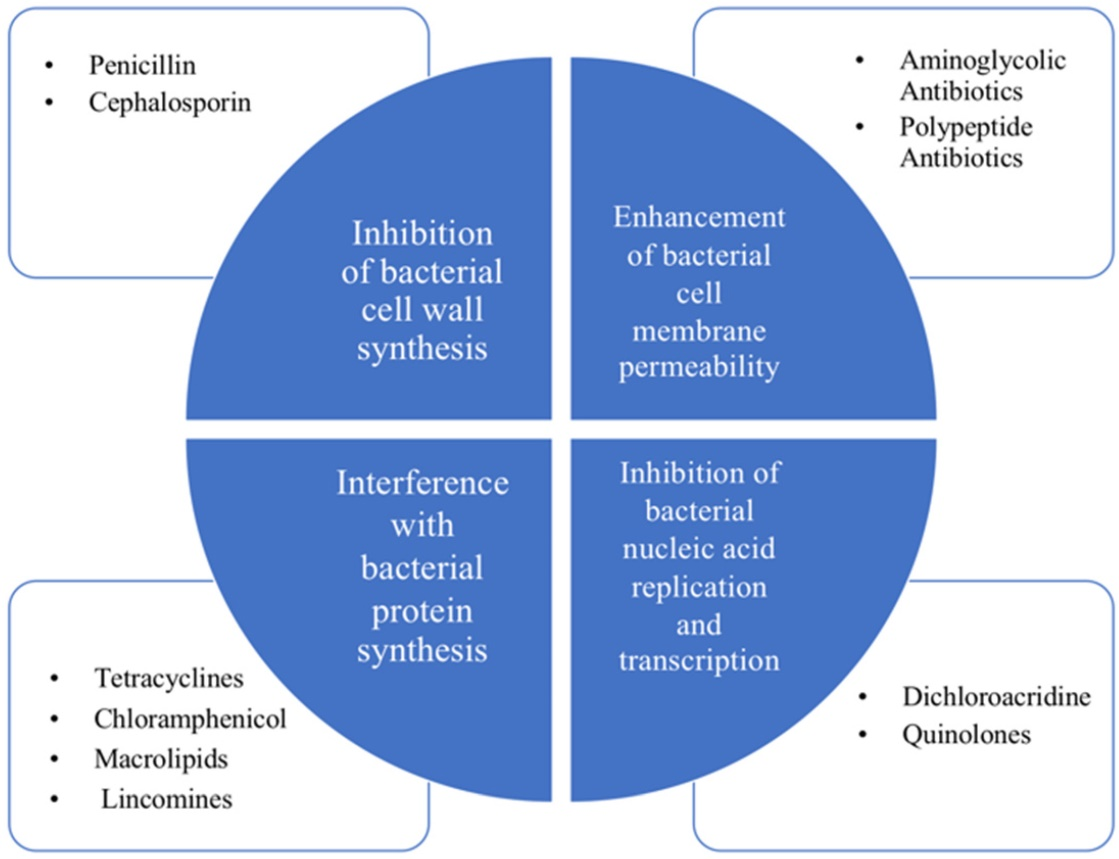
Classification and antibacterial mechanism of antibiotics. The four major mechanisms: inhibition of bacterial cell wall synthesis, enhancement of bacterial cell membrane permeability, interference with bacterial protein synthesis and inhibition of bacterial nucleic acid replication and transcription.

Antibiotic is a kind of medicine that can kill or inhibit the growth of bacteria. It generally refers to the substances produced by bacteria, molds or other microorganisms in the process of life 4. The first breakthrough in antibiotics dates back to 1929 by Alexander Fleming. As the years pass, the antibiotics commonly used at present mainly include β~lactams, aminoglycans, tetracycline, chloramphenicol, macrocyclic lipids, lincomines, polypeptides, rifamycin and quinolones 4. Their bacteriostatic or bactericidal effects mainly include four major mechanisms, which are inhibition of bacterial cell wall synthesis, enhancement of bacterial cell membrane permeability, interference with bacterial protein synthesis and inhibition of bacterial nucleic acid replication and transcription 4. Their bacteriostatic or bactericidal effects mainly rely on four major mechanisms shown in Figure 1, which are inhibition of bacterial cell wall synthesis, enhancement of bacterial cell membrane permeability, interference with bacterial protein synthesis and inhibition of bacterial nucleic acid replication and transcription 8.

Anticancer antibiotics are chemicals produced by microorganisms with anticancer activity 9. They are mainly peptides and anthraquinones, with obvious and effective inhibitory effect on the uncontrolled proliferation, aggressive growth and metastasis of malignant cancers. The classification of anticancer antibiotics mainly includes anthracyclines, mitomycin, bleomycin, actinomycin, guanorycin and endiyne 9, 10. In addition, their anticancer effects are also very complex and effective. Anthracyclines anticancer antibiotics mainly include daunorubicin, doxorubicin, epirubicin and mitoxantrone. Doxorubicin has a broad clinical anticancer spectrum. It is generally used for solid cancers, and is effective against malignant lymphoma, breast cancer, lung cancer, liver cancer, gastric cancer and soft tissue sarcoma, but can also have a good effect on acute leukemia 11, 12. Both daunorubicin and doxorubicin have an anthracyclic plane, which can be inserted between DNA base pairs and tightly bound to DNA, becoming an obstacle of DNA spatial structure, thus inhibiting DNA and DNA-dependent RNA synthesis, and can selectively act on purine nucleosides 13. At the same time, with the effects of cytotoxicity, daunorubicin and doxorubicin can also inhibit the activity of nuclear topoisomerase, interfere with the DNA fracture-reconnection reaction caused by topoisomerase, leading to DNA double strand break, as well as DNA single strand break 14. Mitomycin has anticancer effect on a variety of cancers, also works as a non-specific drug in cell cycle 15. It has a broad anticancer spectrum and a fast curative effect, however, its therapeutic index is low and its toxicity is high. When mitomycin bind to DNA, most of them only act on one strand, and some of them form a cross-link to block the disassembly of DNA double strand, thus destroying the stable double helix conformation of DNA to some extent 15, 16. Bleomycin was the first glycopeptide antibiotic isolated from *Streptomyces rotundus* by metzibinoff of the Japanese institute of microbiology and chemistry 17. It can effectively inhibit the synthesis of DNA, causing the break of single and double strands of DNA 17. Actinomycin is a class of antibiotics containing cyclic peptides, which can be embedded in the groove of the DNA double helix to form a complex with DNA, thereby inhibiting the function of RNA polymerase and ultimately inhibiting RNA synthesis 18. Defuminomycin can form stable complexes with DNA and interfere with DNA template, thus selectively inhibiting RNA synthesis. The effect of endiyne anticancer antibiotics is similar to that of actinomycin anticancer antibiotics.

## The Mechanisms and Rationale for Cancer treatment with Antibiotics

### Proven and potential cellular mechanism for cancer treatment with antibiotics

**Figure 2:**
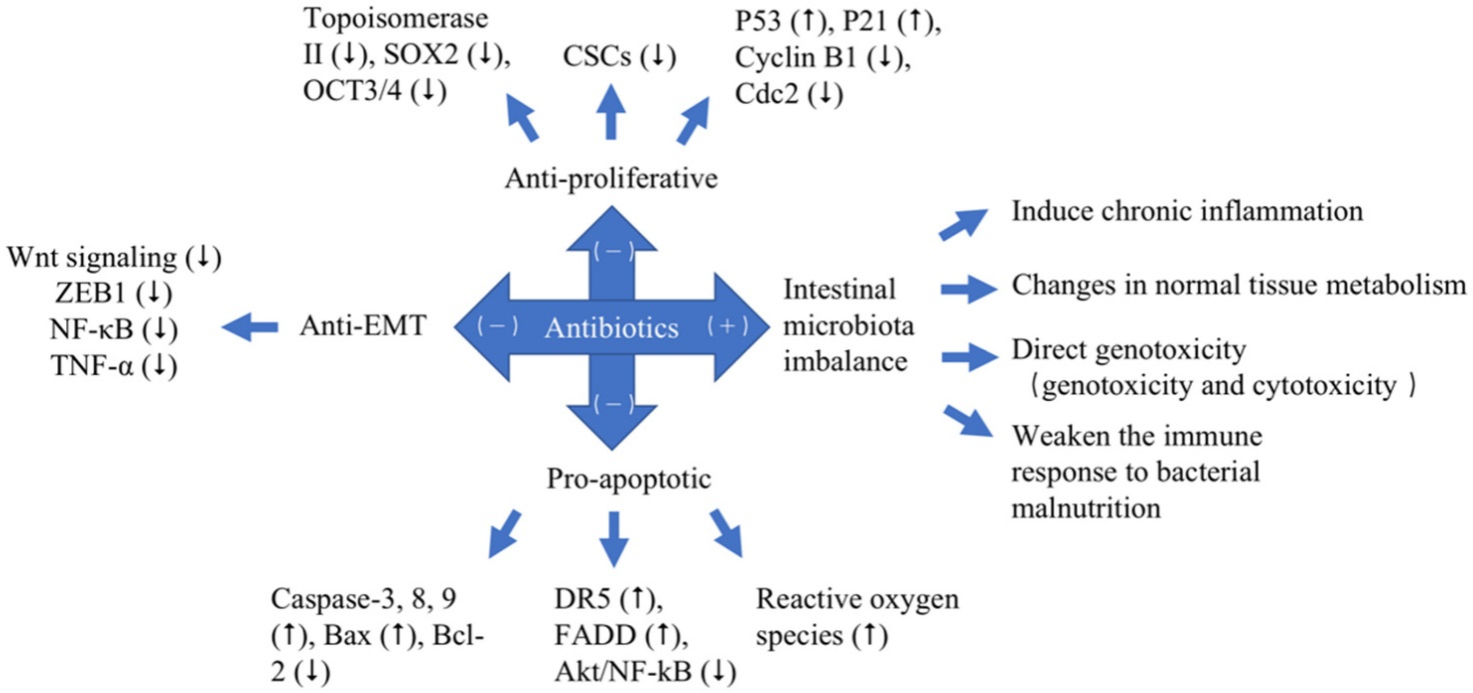
The different effects of antibiotics to cancers with different mechanisms. Pro-cancer (+). Anticancer (-). Up-regulated (⭡). Down-regulated (⭣).

With the rapid development of modern science and technology, especially biomedicine in the 20th century, the understanding of cancer etiology has reached the cellular and molecular levels. According to modern cell biology, cancers are a class of cellular diseases characterized by abnormal cell growth. Since each cancer originates from a single cell, the malignant behavior of cancer cells is transmitted to their progeny through cell proliferation, and cancers are also diseases that involve changes in the structure and function of genetic material (DNA). Meanwhile, the invasive growth and metastasis of cancer cells are also the promotion factors of the occurrence and development of cancer. Anticancer class antibiotics are one of the most important classes of antibiotics, which have their specific inhibitory effects on cancers 19. It can be shown in Table 2 and Figure 2 that anticancer antibiotics have anticancer effects principally through three mechanisms, which are anti-proliferative, pro-apoptotic and anti-epithelial-mesenchymal-transition (EMT).

### Proven and potential molecular mechanism for cancer treatment with antibiotics

On the one hand, anticancer antibiotics can kill cells in the whole proliferation cycle, even G0 phase cells, thereby achieving the anti-proliferation ability of cancer cells by affecting the cell cycle, such an example with cyclinenon-specific drugs (CCNSC) 20. On the other hand, anticancer antibiotics can promote apoptosis of cancer cells by targeting apoptotic gene B cell lymphoma-2 (Bcl-2), apoptotic pro-Bcl-2-associated x (Bax), caspase-3/8/9 and cancer suppressor gene P53, thereby impacting cancer cell apoptosis in patients 21. In addition, anticancer antibiotics can be used as regulatory agents of EMT to inhibit the metastasis of cancer cells and play an anti-metastasis role 22. For example, ciprofloxacin has pro-apoptosis ability while salinomycin can inhibit proliferation and EMT in the development of cancers 23.

Many experts have found that, in particular, cancers tend to be more sensitive to emerging classes of anticancer antibiotics that they have never been exposed to before and that these agents are more effective in inhibiting cancers than those to which the cancer patients have previously been exposed. From the initial anticancer antibiotics such as doxorubicin, epirubicin and mitomycin to the anticancer antibiotics adriamycin, salinomycin and fluoroquinolones discovered in the latest research, the treatment of antibiotics for cancer has become increasingly important.

## The Specific applications of Antibiotics for Cancer treatment

### Adriamycin

Adriamycin is an effective and essential anticancer drug, in particular serving as an indispensable therapeutic ingredient for many childhood cancer patients 24. It is one of the most commonly used anthracyclines in adults and children. The promycin adriamycin was first isolated from the *Streptomyces variety*. Caesius was used as an antibiotic in the 1950s 25. It was not until the 1960s that the drug was first used as a chemotherapeutic 25. To date, more than 2,000 analogues have been developed, but only a few antibiotics have been approved by the FDA for clinical use 24.

Despite its many functions, doxorubicin is known as a DNA implant and produces reactive oxygen species (ROS) through several different cellular mechanisms 26. DOX electrostatically binds to small grooves in DNA fragments 26. In addition to interfering with the synthesis of DNA and macromolecules, adriamycin can also trap topoisomerase II in the normal enzyme activity of topoenzyme II, resulting in DNA double-strand breakage 27. The immediate consequence of the exposure is the up-regulation of p53, a reaction to DNA damage that ultimately leads to programmed cell death. Adriamycin is known to bind to DNA-related enzymes, insert DNA base pairs, and target multiple molecular targets to produce a range of cytotoxic effects 26. For example, it induces activation of various molecular signals from amp-activated protein kinase induces apoptosis (AMPK), which affects the Bcl-2/Bax pathway. By changing the Bcl-2/Bax ratio, the downstream activation of different cystatin proteins can occur, leading to apoptosis 26. Its main role is to inhibit topoisomerases I and II and insert them into DNA to interfere with their helices, eventually inducing programmed cell death 26.

### Bleomycin

Bleomycin is an antibiotic that can be embedded in DNA with iron complexes, causing single-strand and double-strand breaks in DNA to have antibacterial effects 28. In recent years, bleomycin has been used as an effective clinical anticancer drug to treat germ cell cancers and lymphomas as well as squamous cell carcinoma 28. Bleomycin can bind to DNA through its amino terminal peptide, and in the case of oxygen and iron, form DNA-Fe-bleomycin complex, which activates to produce free radicals (hydroxyl radicals) 28. Unfortunately, the development of pulmonary fibrosis has seriously hampered the therapeutic effect of bleomycin. However, the mechanism of pulmonary fibrosis induced by bleomycin, especially the molecular targets of bleomycin, is still unknown.

### Ciprofloxacin

Fluoroquinolones are broad-spectrum synthetic antibiotics that inhibit the synthesis of bacterial DNA 29. Despite their accidental discovery, they are the largest class of antimicrobials in use in the world today. These drugs are widely used in clinical practice to treat urinary, respiratory and gastrointestinal infections, bone, skin or soft tissue infections, and eye diseases 29. They also showed immunoregulatory effects by regulating cytokine production, anti-inflammatory responses, and protection against LPS-induced liver injury 30. The anticancer potential of fluoroquinolones is being studied in a wide range of modules.

Ciprofloxacin is a second-generation fluoroquinolone that is particularly effective against gram-negative bacilli, including *Pseudomonas aeruginosa* 31. The drug has a high bioavailability and a large distribution 31. Ciprofloxacin is widely used in respiratory, urinary and gastrointestinal infections due to its good pharmacokinetic properties and capacity to achieve higher concentrations in tissues than in plasma 32. The anticancer activity of Ciprofloxacin has been demonstrated in human and animal cancer cell lines *in vitro*, such as human bladder cancer cell lines, human colorectal cell lines, hamster ovarian cancer cell lines, and human hepatocellular carcinoma 33, 34. Artur Beberok and Dorota Wrzesniok et al. have shown that the incubation period would be extended to 48 h and 72 h in low concentration (0.01 mM and 0.1 mM) of ciprofloxacin, and that melanoma cells would appear stagnation of S phase of the cycle by cell fluorescence image analysis through mechanisms associated with topoisomerase II inhibition 33, 34. In contrast, Kloskowski et al. demonstrated that ciprofloxacin caused cell stagnation at the G2/M checkpoint in human non-small cell lung cancer 35. At the same time, Hamada H.H. Mohammed et al. showed that derivatives of ciprofloxacin could increase the expression of p53 and p21 and decrease the expression of cyclin B1 and Cdc2 proteins which were an important protein to modulate cell cycle of G2/M in human non-small cell lung cancer cells without any effect on the same proteins expression in other normal cells, which showed dose dependent G2/M arrest in human non-small cell lung cancer cells by flow cytometric analysis 36. Taken together, derivatives of ciprofloxacin induced G2/M phase arrest via p53/p21 dependent pathway and expression of cyclin B1 and Cdc2 proteins. So, ciprofloxacin can exert anti-proliferative effect by regulating the cell cycle process.

Today, fluoroquinolone in promoting the role of cancer cells apoptosis is more and more attention. Artur Beberok et al in 2017 showed that ciprofloxacin induced DNA fragmentation in oligonucleosomes at the highest concentration (1.0 mM) (the alleged presence of sub-g1 fragments) strongly suggesting the induction of apoptosis by mitochondrial dependent pathways, such as disruption of mitochondrial membrane potential 33. One year later, Artur Beberok et al reported that cipfloxacin treatment stimulated the loss of the mitochondrial transmembrane potential via the Bax/Bcl-2-dependent pathway, thus inducing apoptosis in human triple-negative breast cancer MDA-MB-231 cells 34. Oligonucleosomal DNA fragmentation and the elevation of p53 expression were observed in this study, indicating that this late-apoptotic event may be mediated by the p53-dependent pathway 34.

### Dactinomycin

So far, at least 50 kinds of dactinomycin have been found, and only dactinomycin D and C have clinical application value, which is also known as actinomycin 37. Dactinomycin D is a polypeptide antibiotic extracted from the nutrient solution of *Str. Parvul-lus* 38. Its molecules contain a phenoxy ring structure that connects two allelic cyclic peptide chains 38. This peptide chain can interact with deoxyguanine of DNA molecule in a specific way, making dactinomycin D embedded in the groove of DNA double helix, forming complex with DNA, hindering the function of RNA polymerase, inhibiting the synthesis of RNA, especially the synthesis of mRNA, thus inhibiting the occurrence and development of cancer 37, 38. Dactinomycin has a narrow anticancer spectrum and is mainly used for nephroblastoma, chorionic epithelial carcinoma, rhabdomyosarcoma and neuroblastoma 37.

### Daunorubicin

In 1957, researchers in Farmitalia's laboratory extracted pilomycin from *Streptomycin peucetius* in culture 39. Then, in 1963, Di Marco et al. demonstrated the anticancer effect of daunorubicin in a preclinical trial 40. At the same time, French scientist Phome-phouleuc et al. isolated the same substance erythrobicin from the culture solution of *Streptomycin ceruleorubidus* in the laboratory 39, 40. A few years later, Chinese scholars obtained the same strain in the soil of Hebei province, extracted the same substance and named it candimycin 39. Later, all of these similar mycin were uniformly named as daunorubicin. Daunorubicin is a first-line cancer antibiotic, widely used in acute myelogenous leukemia, lymphocytic leukemia and other malignant cancers 39. It can inhibit cancer growth by chimerism between DNA base pairs of cancer cells and tightly binding to DNA, resulting in the obstruction of DNA spatial structure 39.

### Epirubicin

Epirubicin is a new anthracycline antibiotic that can be embedded directly between DNA nucleobase pairs to interfere with the transcription process and prevent the formation of mRNA, thereby inhibiting the synthesis of DNA and RNA 41. In addition, it also can inhibit topoisomerase II. Epirubicin, as a cell cycle nonspecific drug, is effective against a variety of transplanted cancers. It is commonly used for the treatment of breast cancer, malignant lymphoma, soft tissue sarcoma, gastric cancer, malignant melanoma, colon cancer, lung cancer, ovarian cancer and so on 41. But epirubicin has also been shown to inhibit bone marrow, cardiac toxicity, hair loss, mucositis, gastrointestinal tract reactions, high fever, and other adverse reactions 41.

### Gemifloxacin

Gemifloxacin (GMF) is a fluoroquinolone antibiotic that inhibits bacterial DNA gyrase and topoisomerase IV, which not only has anti-proliferative and pro-apoptotic effects, but also has an anti-metastatic activity 42. Tun-Chieh Chen et al. first showed that GMF suppressed the activation of NF-κB, as well as the cell migration and invasion induced by cancer necrosis factor α (TNF-α) 43. Cancer cells in which NF-κB is constitutively active are highly metastatic, and inhibition of NF-κB activity in these cells greatly decreases their invasiveness 43. The transcription factor Snail, one of the target genes of NF-κB, is a key regulatory factor in EMT and cell migration 44. Its expression is elevated in several cancer types, including breast cancer 44. Snail transcriptionally suppresses the adherent junction protein, E-cadherin, by binding to E2-box-type elements within its promoter, resulting in EMT 45. E-cadherin loss and EMT induction have been implicated in the enhancement of metastatic ability and are closely correlated with poor prognosis 45. Tun-Chieh Chen et al. found that GMF had a significant inhibitory effect on Snail expression, which was consistent with the blockade of NF-κB by GMF 43. The inhibition of Snail was directly associated with the restoration of E-cadherin. Overexpression of Snail decreased the anti-migration properties and upregulated E-cadherin in human breast adenocarcinoma cells 43. Their findings also revealed that GMF decreased cancer metastasis by decreasing the localization of Snail in cell nuclei. In conclusion, GMF might be a novel anticancer agent for the prevention and treatment of metastasis in cancer.

### Mitomycin

Mitomycin is an anticancer drug isolated from *Actinomycin* culture, which is effective in multiple solid cancers and is one of the commonly used cycle non-specific drugs 46. The structure of benzoquinone, ulatan and ethylene imine form the three effective groups 46. After the activation of reductase in cells, it acts to depolymerize DNA and antagonize DNA replication. High concentration also inhibits the synthesis of RNA and protein. It is mainly used in late G1 phase and early S phase 47, and also works in acidic and hypoxic conditions 47. The drug resistance is mainly caused by the decrease of membrane permeability and the decrease of intracellular concentration 47, and degradation accelerates the so-called mutation-selection mechanism. Mitomycin includes three anticancer antibiotics: mitomycin A, mitomycin B, mitomycin C (MMC) 46. The mechanism of action is through the formation of double or intra-strand cross-links with DNA in order to inhibit DNA replication and synthesis 48. In addition, MMC-induced addition of oxygen radicals may also contribute to anticancer activity 48. The adverse reaction of this drug is bone marrow suppression, mainly manifested as decreased platelets 48. In addition, drug exosmosis can cause tissue ulcer necrosis, medication with doxorubicin 48.

### Mitoxantone

Mitoxantone is a class of anticancer antibiotics, its structure and anticancer effect is similar to doxorubicin 49. Because Mitoxantone does not have an amino sugar structure, it does not produce free radicals while inhibiting lipid peroxidation, so Mitoxantone is less toxic to the heart 49. Moreover, mitoxantrone can enter the cell and interact with the mitochondria, thus inhibiting the cancer 49.

### Plicamycin

Plicamycin is also known as brilliant-mycin. Antibiotics extracted from *Streptarrryces tanushiensis* or *S. 684* strain in culture 50. Plicamycin has a strong inhibitory effect on a variety of cancers, and its mechanism of action is to bind to DNA, inhibit the synthesis of RNA, and act on cell proliferation at each stage 50. Plicamycin is mainly used in the clinical treatment of testicular embryonal cancer and various malignant cancers caused by hypercalcemia, can also be used for glioma and lymphoma, etc 50. However, the main side effects are gastrointestinal reactions, bleeding, liver, kidney damage and so on 50.

### Salinomycin

Salinomycin, originally used as an antimicrobial agent to kill bacteria, fungi and parasites and to increase the effectiveness of feed for ruminants 51, was isolated from bacteria of the genus *Streptomyces alba* and was produced by a tank fermentation technique originally used as an agricultural antimicrobial substitute 51. This monocarboxylic polyether ionophore is a 751 Da antibiotic, weakly acidic in nature, and acts as an ionophore 51. As an ionophore with strict selective alkali ions and a strong preference for potassium, it interferes with transmembrane potassium potential 52. Salinomycin has shown anticancer property, particularly against many types of cancer stem cells (CSCs), and sensitizes multidrug-resistant human cancer cells 52.

CSCs play an important role in cancer survival, proliferation, metastasis and recurrence 53. In essence, they maintain the vitality of the cancer cell population through self-renewal and unlimited proliferation and enable the metastasis of cancer cells 53. Therefore, the use of CSC as a target for cancer therapy is very important. Unfortunately, CSCs can remain dormant for long periods of time and have multiple drug-resistant molecules that are insensitive to the physical and chemical factors that kill cancer cells 53. Gupta et al. first unveiled that salinomycin selectively killed CSCs by high-through screening method, which showed the activity of Salinomycin toward CSCs was 100 folds higher than the comparatively conventional chemotherapeutic drug 54. At the same time, it was reported that salinomycin could overcome drug resistance in human cancer cells 54. In 2014, Xiao et al reported that salinomycin could significantly inhibit the spheroid-forming capability of non-small cell lung CSCs, reflecting the suppression of self-renewal abilities (20). Years later, Hyun-Gyo Lee, So-Jin Shin et al. showed that salinomycin treatment lessened the expression of sex determining region Y-box 2 (SOX2) and octamer-binding transcription factor 3/4 (OCT3/4) mRNA using reverse transcription polymerase chain reaction and protein levels using western blot analysis 55. CSCs show that the stemness transcription factors, such as SOX2, OCT3/4 and NANOG, play critical roles in stem cell proliferation and self-renewal, so the decrease in these gene expressions indicates the antiproliferative effect of salinomycin on CSCs 55. Some mechanisms of salinomycin targeting CSCs in the treatment of cancers are still under research and are not yet clear, but the therapeutic effect on cancers has been demonstrated in recent years.

Over the past decade, there has been a growing recognition that cancer can be treated by promoting apoptosis. In the process of cell apoptosis, caspase-3, caspase-8, caspase-9, Bcl-2 and Bax are vital factors. Caspase-3, caspase-8, caspase-9 and Bax are pro-apoptotic genes, which can stimulate the apoptosis of human cancer. In contrast, Bcl-2 is anti-apoptotic gene, which can inhibit the apoptosis of human cancer 23. Jin Zhou et al in 2013 reported that the mRNA and protein expression levels of caspase-3, caspase-8, caspase-9 and Bax were effectively up-regulated by salinomycin, while the mRNA and protein expressions of Bcl-2 in cancer cells were down-regulated 21. In their study, they treated colon cancer cells continuously with 5 mol/l of cisplatin and successfully obtained cisp-resistant SW620 cells 21. They then tested specific stem cell markers to see if cisplatin resistant SW620 cells showed dry-like characteristics 21. To discuss the killing effect of salinomycin on cisplatin resistant SW620 cells, they treated cisplatin resistant SW620 cells and protosw620 cells with a certain concentration of salinomycin and observed changes in cell cycle and apoptosis 21. A large number of studies have shown that the proportion of Bcl-2/Bax was reduced in different types of cancer cell apoptosis, indicating that salinomycin can promote the apoptosis of cancer cells 21. In addition, Bidur Parajuli et al also reported in two papers that salinomycin was identified by tetrazolium dye assay to increase the expression of death receptor 5 (DR5) and fas-related proteins with death domain (FADD), and to inhibit Akt/NF-kB induction of cisplatin resistant ovarian cancer cell apoptosis 56, 57. Furthermore, Jin Zhou et al reported that apoptosis of cisplatin-resistant colorectal and prostate cancer cells is achieved through accumulation of reactive oxygen species 56.

The synergistic action of salinomycin and conventional chemotherapy drugs can inhibit the invasion and migration of cancer cells. The transcription factor ZEB1 is known to promote metastasis of cancer and is highly expressed in primary hairline cell lymphoma (MCL) with well-orchestrated active Wnt signaling 22. Sanchez-Tillo E et al in 2014 reported that salinomycin could block Wnt signaling and down-regulate ZEB1, thereby increasing the sensitivity of MCL cells to the cytotoxic effects of gemcitabine, cytarabine and adriamycin 58. Combined with metformin, salinomycin blocked tumor growth factor (TGF)-induced EMT and inhibited EMT-induced cell migration in two non-small cell lung cancer (NSCLC) cell lines A549 and HCC4006 58.

## Pro-cancer effect of Antibiotics in the Course of Treatment

The human gastrointestinal tract is a complex ecosystem, with approximately 10^11^ bacteria per gram faeces 59. These bacteria play an important role in health maintenance and disease development through their involvement in nutrition, pathogenesis and immunology of the host 59. The equilibrium of the normal microbiome acts as a barrier against colonization by potentially pathogenic microorganisms and against uncontrolled overgrowth of microorganisms already present in the gastric intestinal tract, such as yeasts or *Clostridium difficile* 60. Under normal physiological conditions, the balanced state of the microbiome has an immune protective effect, as the cell wall of the dominant bacterial community is the stimulator of certain immune cells. It can activate these cells and stimulate the secretion of antibody, thus improving the body's immunity and resisting the attack of external microbiota 60, 61. In contrast, intestinal microbiota disorder is an important cause of immune function damage and can trigger a systemic inflammatory response, which can, under certain conditions, induce the occurrence of cancer in patients 60, 61.

Multiple lines of modern research suggest that excessive treatment of antibiotics in the treatment of cancer has unintended and, frequently, undesirable consequences. Although it is known that antibiotics have short-term impacts on the human microbiome, recent evidence demonstrates that the impacts of some antibiotics can remain for extended periods of time 62. Administration of antibiotics causes disturbances in the ecological balance between the host and the microorganisms 62. Excessive antibiotic treatment will lead to a series of intestinal dysbacteriosis, reducing the number of* Lactobacillus* and *Bifidobacterium*, as well as the number of harmful bacteria resistant. Researchers have found that the use of antibiotics on premature babies within 7 days of birth significantly lowered the proportion of *Enterobacteriaceae* and reduced the diversity of the microbiome. To eliminate harmful bacteria, antibiotics can also inadvertently remove the beneficial bacteria, which in turn causes intestinal microbiota imbalance 60, 62. In Table 3, these findings outline the possible associations between antibiotic exposure and cancer risk 60, 62. Over the years, a series of antibiotics have been tested, targeting cancer cells and inducing cancer occurrence in the treatment of cancer 62. They may exert their cancer-induced effects via intestinal microbiota imbalance to cause a number of reactions, including effects on inflammatory factors that induce chronic inflammation; changes in normal tissue metabolism; direct genotoxicity, which consists of genotoxicity and cytotoxicity; effecting the efficacy of checkpoint inhibitors and weakening of the immune response to bacterial malnutrition (Figure 2).

### Surgical treatment

Surgical treatment is one of the most effective and common methods for the treatment of cancers. With the exception of malignant cancers of the blood system (such as leukemia, malignant lymphoma), most solid cancers can be treated in the early stages of the disease with surgery. When clinically indicated, the appropriate use of antibiotics can not only prevent or reduce wound infection but can also accelerate wound healing 63. However, as with any form of trauma, the body's immunity and resistance following surgical intervention will be reduced 63. The additive use of antibiotics in such a setting can further weaken the body's immunity through intestinal microbiota disorder, potentially increasing the risk of cancer recurrence 60, 62.

In 2008, Annamari Kilkkinen et al first reported that the use of penicillin predicted an increased risk of cancer, and the relative risks (RRs) with 95% confidence intervals (95% CIs) of the most common colon cancer was 1.15 (1.04-1.26) 64. According to the results, Annamari Kilkkinen supposed that penicillin treatment might promote the development of cancer by affecting intestinal microbiota, which had a major impact on the health of the host 64. Similarly, studies by Ben Boursi et al in 2015 also showed that through nested case-control studies using a large population database from the United Kingdom, past exposure to penicillin might be associated with a modest increase in colorectal cancer (CRC) risk 65. Moreover, Ben Boursi postulated that gut microbiota may have served as an important promoter of CRC formation and that, at the same time, the penicillin therapy could have altered the human microbiota 65. These two independent studies indicate that penicillin affects the composition and function of intestinal flora and immune function.

Under pathological state, the balance of dominant bacterial community and subdominant microbiota is broken, and the intestinal barrier function is damaged. Bacterial structure, function and space position can also be changed, which may eventually cause endogenous infection 60, 62. Many studies have shown that the abnormal intestinal microbiota count or space position could lead to immune function damage 60, 62. In addition, there is strong evidence that indicates that there is cellular immune dysfunction in patients with intestinal microbiota disorder. In terms of humoral immunity, serum immunoglobulin IgA, IgM and IgG levels of patients with intestinal microbiota disorder were lower than those of normal people, indicating that there was also damage of humoral immune function in patients with intestinal microbiota disorder by the use of antibiotics 66.

### Radiotherapy

Cancer radiotherapy is a local treatment of cancers with radiation. About 70% cancer patients need radiation during cancer treatment, and about 40% cancers can be cured by radiation 67. Radiotherapy plays a very important role in the treatment of infantile and vigorous cancer cells, and its role and status in cancer treatment have become increasingly prominent 67. But while radiation destroys and kills cancer cells, it also destroys surrounding normal tissue cells 67.

Simultaneous treatment with antibiotics and radiotherapy is a common way to fight cancers. Interestingly, some studies have shown that tetracycline-induced breast cancer is also related to changes in intestinal microbiota 68, 69. Intestinal microbiota converts phytochemicals, such as glucosinolates, isoflavones, and lignans, to compounds that are biologically active in the human body, which may play an inhibitory role at several points in the carcinogenesis pathway and, specifically, may modulate carcinogen and steroid-hormone metabolism, thereby decreasing breast-cancer risk 68, 69. Adlercreutz H et al and Shapiro TA et al in 1998 reported that tetracyclines treatment disturbs intestinal microbiota and reduces the conversion of glucosinolates, isoflavone glycosides and lignans to biologically active agents 68, 69. Low levels of phytochemicals in urine and serum indicate that the body has fewer chemoprophylactic phytochemical metabolites available, possibly leading to an increased risk of breast cancer 68, 69. Antibiotics have the potential to not only weaken the immune response but can also affect the metabolism of normal tissues through intestinal microbiota disturbances, especially inhibiting the metabolism of plant compounds in normal tissues 60, 62. This has a synergistic effect with the destruction of normal tissues in radiotherapy, which greatly reduces the effect of treating cancers during chemotherapy.

### Chemotherapy

Chemotherapy is one of the most effective ways to treat cancer 70, along with surgery and radiotherapy 70. Surgery and radiotherapy are local treatments that are only effective for cancers at the site of operation, but not for potential metastases or cancers that have already metastasized clinically 63. In contrast, chemotherapy is a systemic treatment. No matter what route is used for drug administration (oral, intravenous and coelial administration, etc.), chemotherapy drugs will spread throughout most organs and tissues of the body through the blood circulation 70. Therefore, chemotherapy is the main treatment for some cancers that tend to spread throughout the body and for advanced stage cancers that have already metastasized 70.

The use of a combination of antibiotics and chemicals is increasingly advocated during chemotherapy. However, the chronic inflammation caused by antibiotics in combination therapy by disrupting the intestinal microbiota is also a major obstacle to chemotherapeutic treatment of cancers 71. At first, it was widely believed by some scientists that tetracycline induced breast cancer by regulating immune inflammatory factors with disorder of intestinal microbiota 71. In 1979, Thong YH et al first reported in healthy human volunteers that tetracyclines and macrolides inhibited mitogen-induced T-lymphocyte proliferation, which might be an important factor in the association between tetracyclines and breast cancer 72. In this way, tetracycline inhibition of T-lymphocytes may limit the anticancer response, resulting in increased cancer detriment and metastatic potential in women who already have breast cancer 72. Decades later, studies by Attur MG et al suggested that tetracyclines increase PGE2 production, which might increase breast-cancer risk through stimulation of aromatase, stimulation of angiogenesis, inhibition of immune surveillance, enhancement of cancerogenic and metastatic potential 73. Furthermore, Coussens LM et al reported tetracyclines could inhibit production of matrix metalloproteinase, which could induce angiogenesis inhibitors and have apoptotic actions, but it was not cleared for clinical trials 74.

Collectively, these studies indicate that antibiotics administered in the course of cancer chemotherapy treatment can lead to intestinal disorders, affect the immune inflammatory factor and induce the inflammation response through different mechanisms (suppress T lymphocyte proliferation, increase the generation of PGE2 and inhibit MMPs), which further cause cancer illness aggravation and greatly hinder the effect of chemical therapy (Table 3).

### Immunotherapy

Immunotherapy is intended to improve the immunogenicity of cancer, by giving the body a sufficient number of functioning immune cells and related molecules, to inspire and strengthen the body's immune response resistance to cancer, thereby improving the sensitivity of the cancer of the anticancer immunity effect, inducing cancer cellular and molecular specificity and non-specific effect outside the body and achieving the eventual goal of cancer eradication with the aid of molecular biology and cell engineering technology 75. Cancer cell immunotherapy has been regarded as the fourth treatment method after surgery, radiotherapy and chemotherapy, with the inclusion of cytokine therapy, immune cell therapy, gene therapy, molecular targeted therapy and antibody therapy 75.

Kashida et al. first reported that quinolones are not only promoters of liver cancers, but also liver cancer initiators based on *in vivo* alkaline single-cell gel electrophoresis (comet) test and two-stage studies of liver cancer in mice 76. Years later, Tadashi Itoh et al chose four old quinolone [NA, PPA, OA and pyrazole and acid (PA)] and four new quinolone [(ENX), according to the north of sand OFLX, head and CPFX] to study their effects on DNA, and put forward the possibility of the head and CPFX genetic toxicity potential from DNA replication *in vitro* by its inhibition of topoisomerase II activity, rather than from base adduct formation 77. A recent study of antibiotics and immunotherapy suggests that the use of antibiotics can affect the outcome of immunotherapy in patients 78. The results showed that the median overall survival of all immunotreated patients was 14.6 months, including a median survival of up to 26 months for those who did not receive broad-spectrum antibiotics within 30 days of immunotherapy 78. Even more surprising, a subset of patients who received broad-spectrum antibiotics during immunotherapy had a median overall survival of only 2 months. This may occur because antibiotics, when used in combination with immunotherapy, can produce direct immunotoxicity, genotoxicity or cytotoxicity by disrupting the intestinal microbiota, which can inhibit the immunotherapy for cancer 78.

In addition, antibiotics can also affect the therapeutic effect of immunocheckpoint inhibitors by causing intestinal flora disorder in the course of immunotherapy. In 2008, Bertrand Routy showed in an article that the richness of some intestinal flora could promote the anti-tumor effect of the immunocheckpoint inhibitors represented by PD-1 / PD-L1 and CTLA-4 79. However, antibiotic therapy significantly reduced the anti-tumor effect and survival time of mice treated with PD-1 monoclonal antibody alone or combined with CTLA-4 monoclonal antibody by mediating intestinal flora disorder 79. Moreover, multiple courses or long-term use of antibiotics had a greater impact on the efficacy of checkpoint inhibitor therapy than short-term use of antibiotics 79. Therefore, the side effects of anticancer antibiotics in immunotherapy should not be ignored.

## Summary of Primary Beneficial Effects of Cancer Treatment with Antibiotics

Nowadays, antibiotics have been widely recognized in the treatment of cancers. When antibiotics are compared with traditional cancer treatments, such as surgery, radiotherapy, chemotherapy, immunotherapy and targeted therapy, the main performance is to improve the prognosis and reduce the side effects of antibiotics. And the brief description of this is as follows.

In the process of surgical treatment of cancer, although some cancers appear to be limited, but there are already cancer cells into the blood circulation, surgery can only cut out the local cancer, but not the cancer cells which have spread to the blood circulation. As a result, cancer surgery may often be followed by systemic drug therapy with antibiotics to remove the cancer and minimize the risk of recurrence.

Similarly, chemoradiotherapy reduce the number of cancer cells to achieve the therapeutic effect. However, after radiotherapy, most of the cancer patients will relapse after a period of time, which is also a difficult problem to be resolved in the current research of cancer biology, the main reason being the presence of CSCs 80. With the traditional treatment of cancer, most of the cancer cells are eliminated, but if a small percentage of the total number of CSCs are still present, the recurrence of the cancer will occur even after most of the other cancer cells have been eliminated 80. Thus, eradicating cancer requires eliminating CSCs first. Many studies have shown that the use of anticancer antibiotics can kill cancer stem cells in a special way through high-throughput screening, which has a high inhibitory effect on cancer recurrence, and thus successfully reduce the recurrence rate of cancer 52.

The proliferation rate of cancer cells is usually higher than that of the normal cells from which they came 81. Fast-growing cancers (such as acute leukemia and choriocarcinoma) which have high rates of proliferation, are sensitive to anticancer drugs and can even be cured with chemotherapy 81. One major problem, however, is that the cells lack physical space and nutrients due to competition, so cancer cells typically slow down. Slow-growing cancers, such as most solid cancers, including liver, lung, and stomach cancers, have low proliferation rates, low drug sensitivity, and poor efficacy 81, 82. Even for cancers with high proliferation rates, G0 cells are rare, so after conventional anticancer drugs kill the proliferating cells, the G0 cells can proliferate again. Anticancer antibiotic is a new type of anticancer chemotherapy drug. Current studies have shown that the anticancer antibiotic is CCNSC, which can kill cells in the entire proliferation cycle, including G0 stage cells, and further enhance the sensitivity of cancer cells to fight against cancer antibiotics, so as to achieve the effect of inhibiting cancer cells more effectively 20.

Immunotherapy is currently a popular way to treat cancers, and its effective time is about 3 months, so the earlier the intervention, the better. At this point, adjuvant antibiotics can improve the immune function of the body, launch an attack on cancer, improve the overall situation, improve the treatment effect, and effectively prevent the recurrence and metastasis of cancer 83. However, once the terminal stage of cancer is reached, the immune function of the patient is too low, and the immunotherapy is slow to take effect, and the therapeutic effect is often poor. So, the combination of immunotherapy and antibiotics is getting more and more attention. Compared with immunotherapy alone, the efficacy of antibiotics combined with it was better 83.

The clinical application of targeted drugs has indeed brought good news to patients with terminal diseases, at least improving the quality of life of patients and extending the survival period. But current "targeted drugs" are expensive and need to be taken every day. And it should be taken for more than one month. If it is effective, it should be taken orally for a long time until the drug is resistant, which causes a great burden on the patient's family. Moreover, targeted therapy does not have the effect of radical cure and is not effective for all cancers and all patients, which is also its great disadvantage.

## Summry of Primary Adverse Effects of Antibiotics

**Figure 3:**
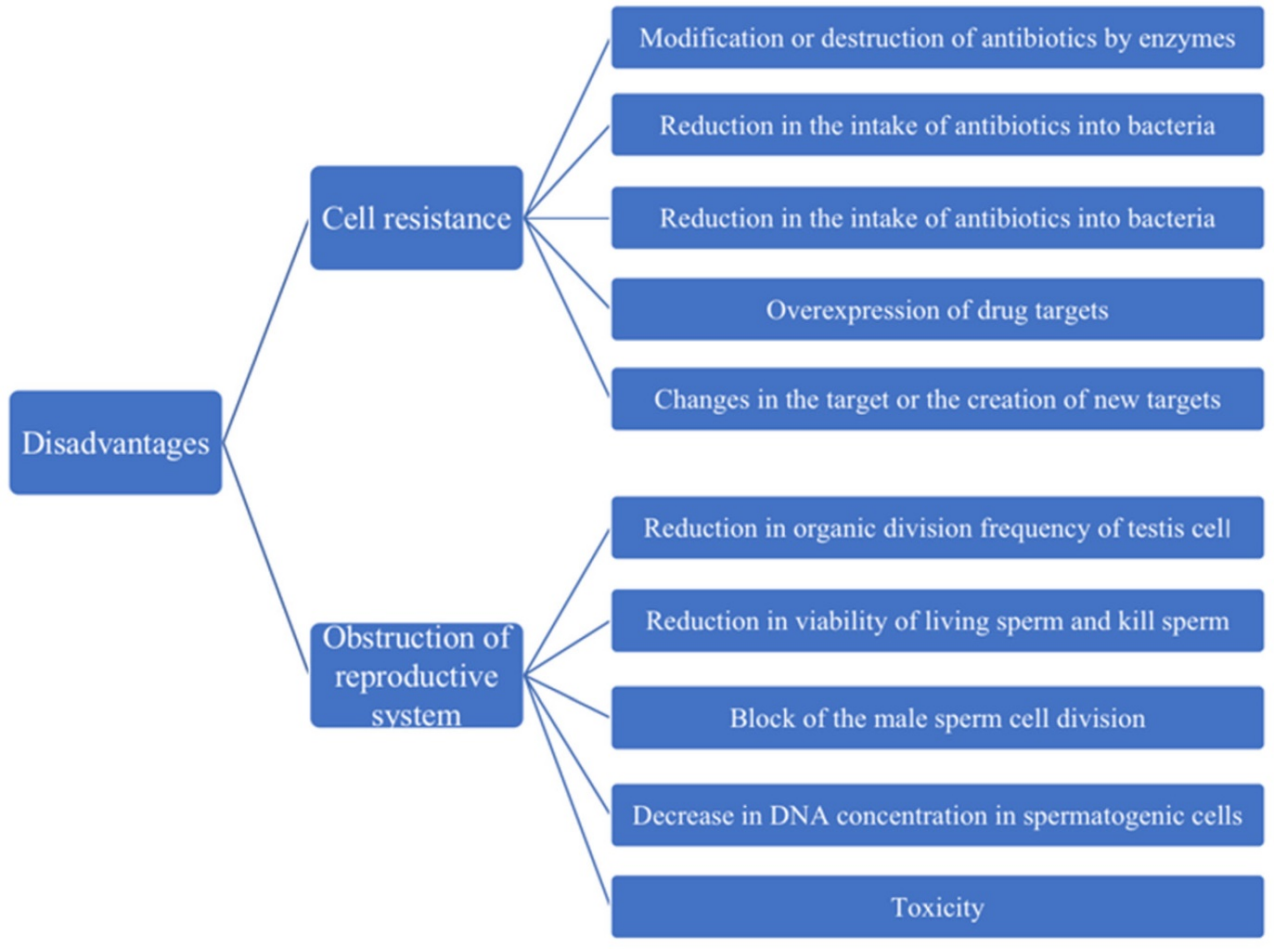
The disadvantages of antibiotics in the treatment of diseases. The major aspects of disadvantages are cell resistance and obstruction of reproductive system.

During the use of antibiotic, bacteria are increasingly resistant to it, so the disadvantages of antibiotic treatment are also slowly becoming apparent 84. Antibiotic resistance can be inherent or acquired through the process of gene mutation or gene transfer. Genetic information encoding these mechanisms can appear through random spontaneous mutations or can be transmitted through genes 84. According to many researches, it is divided into 5 categories: the modification or destruction of antibiotics by enzymes; reduction in the intake of antibiotics into bacteria; increase of antibiotics from bacteria active outward excretion; changes in the target or the creation of new targets and overexpression of drug targets 85-87. Therefore, the resistance of other microorganisms to mutations in the presence of certain antibiotics is more easily selected, and the use of the antibiotics should be carefully managed (Figure 3).

In addition to developing antibiotic resistance to bacteria, frequent use of antibiotics can also lead to male infertility by decimating a man's sperm, greatly reducing his fertility (Figure 3). The impact of these antibiotics on the reproductive system has been questioned by experts for years. In 1995, e. d. Gol 'dberg, administered a single dose of the anthracyclines antibiotic pharmorubicin at the maximum permissible dose in an experiment, found destructive testicular changes and spermatogenic disorders in Wistar rats one to three months later. Moreover, one month after injection, the number of major lethal mutations increased, indicating that the anthracycline antibiotic panerythromycin had a delayed toxic effect on the morphological and functional status of the reproductive system and the genetic apparatus of the sex cells in male rats 88. With the widespread use of antibiotics, the harm of antibiotics to the reproductive system is becoming more and more obvious; in particular, the influence on male reproductive ability cannot be ignored. Studies have shown that macrolidene antibiotics (such as erythromycin, spiramycin, medemycin, etc.) could reduce the organic division frequency of testicular cells, kill sperm, and significantly reduced the viability of living sperm 89. In 2007, McClusky LM found that the use of gentamicin in large quantities could make the male sperm cell division blocked, the DNA concentration in spermatogenic cells decreased, resulting in the spermatogenic process to stop 90. Similarly, Morice Philippe in 2007 found that there would be fertility in women after cancer treatment because tetracycline drugs are toxic to the gonads 91. These experimental results showed in Figure 3 that antibiotics were not only destructive to male reproductive system, but also harmful to female reproductive system, which was also a problem that cannot be ignored for us.

Therefore, we can regulate the use of antibiotics in order to reduce the resistance of bacteria. If infection occurs, the type of bacteria causing the infection should be identified as soon as possible, and the number of combinations of drugs should be reduced as far as possible. At the same time, patients should be actively urged to take medicine according to the prescribed dosage and course of treatment.

## Future Directions

Antibiotics play key role in the treatment of diseases, helping human beings to reduce the mortality rate of various infections, and significantly improving people's quality of life and life expectancy 92. Moreover, antibiotics have been widely used as adjuvant drugs in the process of surgical treatment, radiotherapy, chemotherapy and immunotherapy of cancers due to their anti-infection and anticancer effects 93. However, antibiotic resistance and its harm to the reproductive system are of increasing concern. At the same time, antibiotics can also reduce the effectiveness of surgery, radiation, chemotherapy and immunotherapy in combination, via leading the disorder of intestinal microbiota 60, 62.

In our previous studies, orally taking of probiotics drugs during radiotherapy, chemotherapy and surgery in patients can significantly reduce their side effects by improving the disorder of intestinal microbiota 94, 95. Other works also indicated that dietary supplements (prebiotics) 96 or prebiotics 97 could selectively stimulate the growth and activity of internal probiotics to reduce the disorder of intestinal microbiota. As we known, probiotics in the intestinal tract (such as *Bifidobacterium* and *Lactobacillus*, etc.) are an indispensable element of human health and play a certain protective role in the intestinal tract 98, and the decrease of probiotics was the direct reason for intestinal microbial disorder 98. Considering that the pro-cancer effects of antibiotics are mainly caused by their negative effect on intestinal microbiota, it is therefore a wise choice to reduce the side effect of antibiotics in cancer treatments via taking probiotics and prebiotics.

## Figures and Tables

**Table 1:** Classification of anticancer antibiotics

	Antibiotics	Mechanism of action	Clinical application
Anthracycline	Daunorubicin	Insert into the base pairs of DNA and lead to the obstruction of the spatial structure of DNA	Acute myeloblastic leukemia Acute lymphocytic leukemia;
	Adriamycin	Insert of the double helix into the DNA and inhibit the action of the DNA polymerase	Malignant lymphoma Breast cancer Lung cancer;
	Epirubicin	Interfere with the transcription process and prevent mRNA formation	Leukemia Malignant lymphoma Myeloma;
	Mitoxantone	Insert of the double helix into the DNA and inhibit the action of the DNA polymerase	Malignant lymphoma Breast cancer Acute leukemia;
	Pirarubicin	Insert of the double helix into the DNA and inhibit the action of the DNA polymerase	Lung cancer;
None anthracyclines	Mitomycins	Bind to the DNA and prevent the DNA from breaking apart	Digestive tract carcer Cervical cancer;
	Bleomycins	Cause the DNA to double strand break	Esophagus cancer Cervical cancer Lung cancer;
	Pingyangmycin	Cause the DNA to double strand break	Prostatic cancer;
	Actinomycins	Inhibit the function of RNA polymerase and synthesis of mRNA	Neuroblastoma Nephroblastoma;
	Mithramycin	Inhibit the synthesis of DNA and RNA	Encephalophyma Malignant melanoma;

**Table 2:** Anticancer activity of antibiotics with their mechanisms of action*

S.No.	Antibiotics	Type of Cancer Cell	Mode of Anticancer Activity	Mechanism of Action	Ref
1	Salinomycin	―――	Anti-proliferative	Kills CSCs	54
		Lung cancer cells	Suppression of self-renewal abilities	Inhibit the spheroid-forming capability of non-small cell lung CSCs	20
		―――	Anti-proliferative	mRNA expression of SOX2 (⭣), OCT3/4 (⭣)	55
		Colon cancer cells	Pro-apoptotic	mRNA and Protein expression levels of caspase-3 (⭡), caspase-8 (⭡), caspase-9 (⭡), Bax (⭡), Bcl-2 (⭣)	21
		Ovarian cancer cell	Pro-apoptotic	The expression of DR5 (⭡), FADD (⭡), Akt/NF-kB (⭣)	56, 57
		Colorectal and Prostate cancer cells	Pro-apoptotic	Accumulation of reactive oxygen species	56
		Mantle cell lymphoma (MCL) cells	Anti-Epithelial-Mesenchymal-transition (EMT)	Wnt signaling (⭣), ZEB1 (⭣)	22
2	Ciprofloxacin	Melanoma cells	S-phase arrest	Topoisomerase II inhibition	33, 34
		Human non-small cell lung cancer	G2/M checkpoint arrest	The expression of p53 (⭡), p21 (⭡), cyclin B1 (⭣), Cdc2 (⭣)	35, 36
		―――	Disruption of mitochondrial membrane potential	Oligonucleosomal DNA fragmentation, p53 (⭡)	33
		Human triple-negative breast cancer MDA-MB-231 cells	Disruption of mitochondrial membrane potential	Bax/Bcl-2-dependent pathway (⭡)	34
3	Gemifloxacin	Human breast adenocarcinoma cells	Inhibit the Snail expression, the localization of Snail in cell nuclei (⭣)	The activation of NF-κB (⭣), TNF-α (⭣)	43
4	Doxorubicin	―――	Binds to small grooves in DNA fragments	Produces reactive oxygen species (ROS)	26
		―――	Trap topoisomerase II	DNA double-strand breakage, p53 (⭡)	27
		―――	Activation of various molecular signals from AMPK (⭡)	Bcl-2/Bax ratio (⭣)	27
5	Mitomycin	―――	G1-phase and early S-phase arrest	The synthesis of RNA and protein (⭣)	47
		―――	Form double or intra-strand cross-links DNA	Replication and Synthesis of DNA (⭣)	48

*Up-regulated (⭡). Down-regulated (⭣).

**Table 3:** Pro-cancer activity of antibiotics with their mechanisms of action in different therapy methods via intestinal microbiota imbalance*

Therapy methods	Antibiotics	Type of Cancer Cell	Mode of Anticancer Activity	Mechanism of Action	Ref
Surgical treatment	Penicillin	Colon cancer	Abnormal intestinal microbiota count or space position	Immune function damage, the levels of IgA (⭣), IgM (⭣), IgG (⭣)	65
Radiotherapy	Tetracycline	Breast cancer	Disturb intestinal microbiota	The conversion of glucosinolates, isoflavone and glycosides to biologically active agents (⭣)	68, 69
Chemotherapy	Macrolides	Breast cancer	Disorder of intestinal microbiota	Regulate immune inflammatory factors, induce chronic inflammation	72
	Tetracyclines & Macrolides	Breast cancer	Disorder of intestinal microbiota	Mitogen-induced T-lymphocyte proliferation (⭣)	72
	Tetracyclines	Breast cancer	Disorder of intestinal microbiota	Production of PGE2 (⭣)	71, 73
	Tetracyclines	―――	Disorder of intestinal microbiota	Production of matrix metalloproteinase (⭣)	74
Immunotherapy	Quinolones	Liver cancers	Disrupt the intestinal microbiota	Production direct immunotoxicity (⭡), genotoxicity (⭡), cytotoxicity (⭡)	76, 77

*Up-regulated (⭡). Down-regulated (⭣).
